# Bio-Guided Isolation of Methanol-Soluble Metabolites of Common Spruce (*Picea abies*) Bark by-Products and Investigation of Their Dermo-Cosmetic Properties

**DOI:** 10.3390/molecules21111586

**Published:** 2016-11-21

**Authors:** Apostolis Angelis, Jane Hubert, Nektarios Aligiannis, Rozalia Michalea, Amin Abedini, Jean-Marc Nuzillard, Sophie C. Gangloff, Alexios-Leandros Skaltsounis, Jean-Hugues Renault

**Affiliations:** 1 Institut de Chimie Moléculaire de Reims, UMR CNRS 7312, SFR CAP’SANTE, UFR de Pharmacie, Université de Reims Champagne-Ardenne, Reims 51687, France; amin.abedini@univ-reims.fr (A.Ab.); jean-marc.nuzillard@univ-reims.fr (J.-M.N.); jh.renault@univ-reims.fr (J.-H.R.); 2 Division of Pharmacognosy and Natural Products Chemistry, School of Pharmacy, University of Athens, Panepistimioupolis, Zografou, Athens 15771, Greece; aligiannis@pharm.uoa.gr (N.A.); rozaliamich@pharm.uoa.gr (R.M.); skaltsounis@pharm.uoa.gr (A.-L.S.); 3 Biomatériaux et Inflammation en Site Osseux, EA 4691, SFR CAP-Santé, UFR de Pharmacie, Université de Reims Champagne Ardenne, Reims 51100, France; sophie.gangloff@univ-reims.fr

**Keywords:** dermo-cosmetic agents, Centrifugal Partition Chromatography, ^13^C-NMR dereplication, *E*-astringin, taxifolin, tyrosinase, elastase and collagenase activity

## Abstract

Common spruce (*Picea abies* L.) is a fast-growing coniferous tree, widely used in several countries for the production of sawn wood, timber and pulp. During this industrial exploitation, large quantities of barks are generated as waste materials. The aim of this study was the bio-guided investigation and the effective recovery of methanol-soluble metabolites of common spruce bark for the development of new dermo-cosmetic agents. The active methanol extract was initially fractionated by Centrifugal Partition Chromatography (CPC) using a triphasic solvent system in a step-gradient elution mode. All resulting fractions were evaluated for their antibacterial activity, antioxidant activity and their capability to inhibit tyrosinase, elastase and collagenase activity. In parallel, the chemical composition of each fraction was established by combining a ^13^C-NMR dereplication approach and 2D-NMR analyses. As a result, fourteen secondary metabolites corresponding to stilbene, flavonoid and phenolic acid derivatives were directly identified in the CPC fractions. A high amount (0.93 g) of *E*-astringin was recovered from 3 g of crude extract in a single 125 min run. *E*-Astringin significantly induced the tyrosinase activity while *E*-piceid, taxifolin, and taxifolin-3′-*O*-glucopyranoside exhibited significant anti-tyrosinase activity. The above compounds showed important anti-collagenase and antimicrobial activities, thus providing new perspectives for potential applications as cosmetic ingredients.

## 1. Introduction

*Picea abies* (L.) Karst, (common spruce or Norway spruce) is a large evergreen coniferous species native to Northern, Central and Eastern Europe. This fast-growing tree plays an important economical role in several countries where its wood is widely used for the production of sawn timber and pulp. During this process large quantities of barks are generated as waste materials and usually recycled for energy production [1]. However, the barks of several trees contain high amounts of bioactive components which are involved in chemical defense against pathogens found in woody plants [2]. In addition to plant disease resistance, these compounds have numerous biological activities that could find applications in human health. Thus, this large amount of bark wastes represents an interesting source of high-value chemicals giving added value to this energy resource [3].

Norway spruce bark is a widely studied material with numerous research works regarding the extraction, chemical composition and bioactivity of extracts and purified compounds. The nonpolar bark extract contains high amount of abietane diterpenes, a category of bioactive compounds which is mainly found in coniferous tree resins. Dehydroabietic acid is the main component of the abietane diterpene content with a plethora of bibliographic references regarding its antibacterial and antifungal properties [4]. On the other hand, several phenolic compounds including stilbenes, lignans, flavonoids, and tannins have been referred in hydrophilic bark extracts [5,6]. These compounds are of high interest, with numerous biological properties and applications in food and pharmaceutical industry [7]. Particular attention has been paid to stilbene glucosides which are highly concentrated in spruce bark and have significant antioxidant and protective properties. *E-*Astringin and *E-*isorhapontin are usually the major compounds of these extracts, while *E-*piceid, stilbene aglycones and several dimers of stilbene glucosides have also been identified [8]. Several works have demonstrated the influence of the tree age, extraction method and distribution of chemicals within bark on the yield and activity of extracts as well as on the amount of produced stilbene glucosides and phenolic compounds [9,10,11]. Despite the high number of references regarding spruce bark bioactive compounds, there is a lack of information concerning the effective recovery and purification of these high-value compounds in sufficient amount for a potential industrial exploitation.

In a recent study, ten barks from deciduous and coniferous tree species from north-east France were investigated for their dermo-cosmetic potential. The methanolic bark extract of *Picea abies* exhibited significant antimicrobial, antioxidant, antielastase and anticollagenase activities [12]. In the present work, this bioactive methanol extract was fractionated by Centrifugal Partition Chromatography (CPC) and the resulting fractions were evaluated for their antibacterial and antioxidant activity as well as for their capacity to inhibit tyrosinase, elastase and collagenase activities. Moreover, the chemical composition of each fraction was established by NMR analyses in order to determine which compounds were involved in the observed activities. Finally, five pure compounds, present in the active fractions, were tested in the same assays and interesting information were obtained regarding their potential use as cosmetic ingredients.

## 2. Results and Discussion

### 2.1. Fractionation of Crude Methanol Extract by CPC

In a single 125 min run, 3 g of the active methanol extract were fractionated by CPC using a triphasic solvent system in a step-gradient elution mode. CPC is a solid support-free separation technique involving the distribution and the transfer of solutes between at least two immiscible liquid phases according to their distribution coefficient. The column design used for this work, with only 231 partition cells (FCPE300^®^), offers the possibility to rapidly fractionate multi-gram amounts of natural mixtures and thus to collect fractions in sufficient quantities for further chemical analyses and biological assays. In addition, the use of a triphasic solvent system composed of *n-*heptane, M*t*BE, CH_3_CN, and water was already reported as an efficient method to fractionate natural compounds from barks in a wide range of polarity [13].

**Figure 1:**
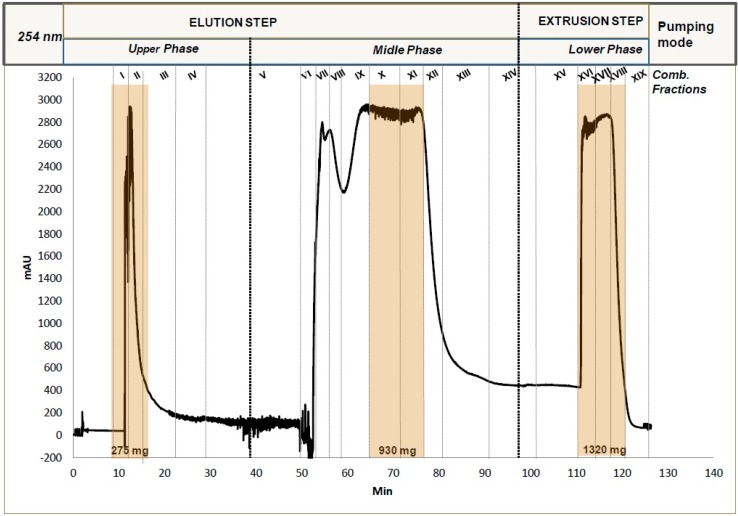
Step-gradient CPC chromatogram of 3 g crude methanol extract of *P. abies* bark at 254 nm; Stationary phase: lower phase of the triphasic system; Mobile phase; upper and middle phases for the elution step and lower phase for the extrusion step, all in ascending mode.

The experiment lasted approximately two hours while UV (Figure 1) and TLC chromatograms (Supplementary Materials, Figure S1) showed a successful fractionation of the methanol extract of *P. abies* bark. The first peak of the UV CPC chromatogram corresponds to the less polar compounds of the extract, which were eluted during the first elution step with the *n-*heptane-rich upper phase of the triphasic solvent system as the mobile phase. Then, the moderately polar compounds were eluted from 50 to 90 min (main peak of the chromatogram corresponds to stilbenes) during the second step of the stepwise gradient when the middle phase was used as the mobile phase. Finally, the last peak of the CPC chromatogram corresponds to the most polar components of the extract obtained from 110 to 125 min during the extrusion step.

### 2.2. Chemical Characterization of CPC Fractions by ^13^C- and 2D-NMR Analysis 

**Figure 2:**
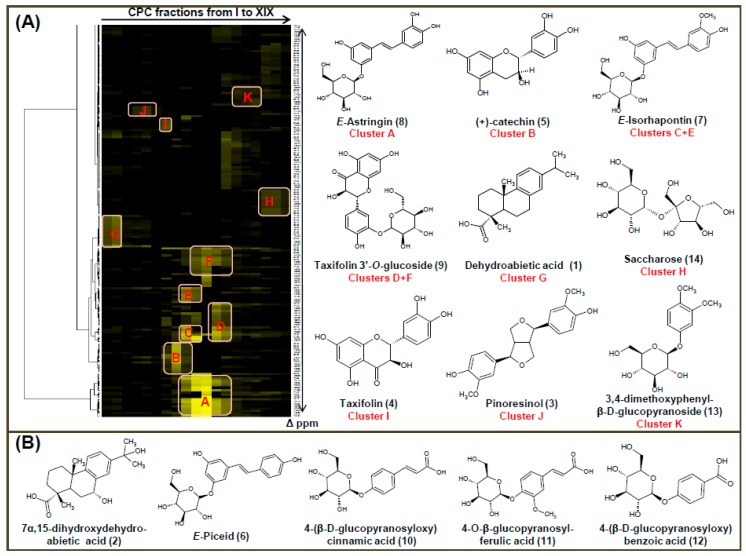
(**A**) ^13^C-NMR chemical shift clusters (yellow color) obtained by applying HCA on CPC fractions of *P. abies* bark extract and the chemical structures of nine major compounds identified by database analysis of the marked clusters (clusters A to K). (**B**) Chemical structures of five minor compounds identified on CPC fractions by 2D-NMR analysis.

The major compounds contained in the CPC fractions were firstly identified by using a recently developed ^13^C-NMR-based dereplication procedure. All CPC fractions (from I to XIX) were analyzed by ^13^C-NMR spectroscopy. Automatic peak picking and alignment of ^13^C-NMR signals across spectra of the fraction series resulted in a table with 19 columns (one per fraction) and 571 raws (one per chemical shift bin containing at least one signal in at least one fraction). By applying hierarchical clustering analysis on the lines of this dataset, statistical correlations between ^13^C-NMR resonances belonging to individual molecular structures within the fractions were readily visualized as well-defined chemical shift clusters colored in yellow in front of the corresponding dendrograms. Figure 2 shows the HCA correlation heat map. After entering the values of each chemical shift belonging to the same cluster into the local database containing ^13^C-NMR data of natural compounds including known metabolites from *Picea abies*, nine compounds were directly identified, including one diterpene, dehydroabietic acid (**1**), three flavonoids, (+)-catechin (**5**), taxifolin (**4**) and taxifolin-3′-*O*-glucoside (**9**), two stilbene glycosides, *E-*astringin (**8**) and *E-*isorhapontin (**7**), one lignan, pinoresinol (**3**), one phenolic derivative, 3,4-dimethoxyphenyl-β-d-glucopyranoside (**13**), and saccharose (**14**) (Figure 2A). The chemical structures of identified compounds were confirmed by HRMS analysis. Dehydroabietic acid is a common abietane diterpene encountered in *Pinus* resins. It was previously reported in the needles, resin and barks of *P. abies* [14]. Pinoresinol, (+)-catechin, taxifolin, taxifolin-3′-*O*-glucoside, *E*-astringin and *E*-isorhapontin are present in several tree species, including *P. abies*. Particularly, stilbene derivatives have been reported as the main secondary metabolites of the polar bark extract of common spruce [15]. 3,4-Dimethoxyphenyl-β-d-gluco- pyranoside has been previously reported in root bark of *P. abies* [15].

Further 2D-NMR analyses of selected fractions led to the identification of five additional metabolites, which were present in the extract as minor compounds (Figure 2B). These compounds were unambiguously identified as 7α,15-dihydroxydehydroabietic acid (**2**) (Fr.IIIFr.IV), *E*-piceid (**6**) (fraction IX), 4-(β-d-glucopyranosyloxy)cinnamic acid (**10**) (Fr.XIII–Fr.XV), 4-*O*-β-glucopyranosyl-ferulic acid (**11**) (Fr.XIV–Fr.XV) and 4-(β-d-glucopyranosyloxy)benzoic acid (**12**) (Fr.XIV–Fr.XVI). The proposed structures were verified by HRMS analysis.

7α,15-Dihydroxydehydroabietic acid was present as a minor compound in Fr.III and Fr.IV. This abietane diterpene has been previously identified in the aerial parts of *Abies georgei* Orr [16] and buds of *Pinus banksiana* Lamb together with its 7β-isomer, [17] while its 4-*epi*-isomer has been referred in stems of *Illicium jiadifengpi* [18] and air-dried fruits of *Juniperus polycarpus* var. *seravschanica* [19]. To our knowledge, this is the first report of the presence of this compound in the genus *Picea*. *E*-Piceid was recovered in Fr.IX together with the stilbene derivatives *E*-astringin and *E*-isorapontin and have been previously reported in many barks, including *P. abies*. Regarding the phenolic acid glucosides, 4-(β-D-glucopyranosyloxy) cinnamic acid and 4-(β-d-glucopyranosyloxy) benzoic acid have been previously reported in *P. abies* [15] while 4′-*O*-β-glucopyranosylferulic acid has been referred only in *P. glauca* [20].

The combination of ^13^C-NMR dereplication with ^1^H and 2D-NMR analyses led to the characterization of the main chemical content of each CPC fraction (Table S1). This qualitative analysis helped us to better associate the activity of each fraction with its components. For instance, the first one contained an important amount of unidentified fatty compounds (fractions I–III) or unsaturated fatty acids (fractions IV–Fr.VI) while the last fractions (XVI–XIX) contained high amount of tannins. This information was taken into account in order to avoid incorrect assessments regarding the activity of components.

It is important to note here that the main compound of the methanol extract is *E*-astringin and was mainly recovered in fractions Fr.X and Fr.XI. The chemical analysis of this fractions revealed that *E*-astringin was present as the major compound (NMR purity > 83%), while taxifolin-3′-*O*-glucoside was also present in small quantities. Both fractions (Fr.X and Fr.XI) yielded approximately 30% m/m of the crude extract highlighting the potential for high volume production of the bioactive *E*-astringin directly from the crude extract by using CPC technology. The addition of one more single step in the separation procedure (Sephadex LH-20 column chromatography) led rapidly to the recovery of high purity (NMR purity > 95%) *E*-astringin and taxifolin-3′-*O*-glucoside (Figure S2).

### 2.3. DPPH Radical Scavenging Activity of CPC Fractions 

All fractions containing phenolic compounds (Fr.VI–Fr.XVIII) exhibited an important radical scavenging capacity (Figure S3). Fractions enriched in tannins (Fr.XVII and Fr.XVIII) were the most active with 54.4% and 62.2% inhibition of the DPPH radical when tested at 25 μg/mL, followed by Fr.XIII–Fr.XVI which contain mainly phenolic acid derivatives. Moreover the fraction enriched in (+)-catechin (Fr.VIII) also exhibited an important radical scavenging activity. The antioxidant properties of these phenolics have been well studied with numerous bibliographic references [21].

### 2.4. Tyrosinase Inhibitory Activity of CPC Fractions and Pure Compounds

**Figure 3:**
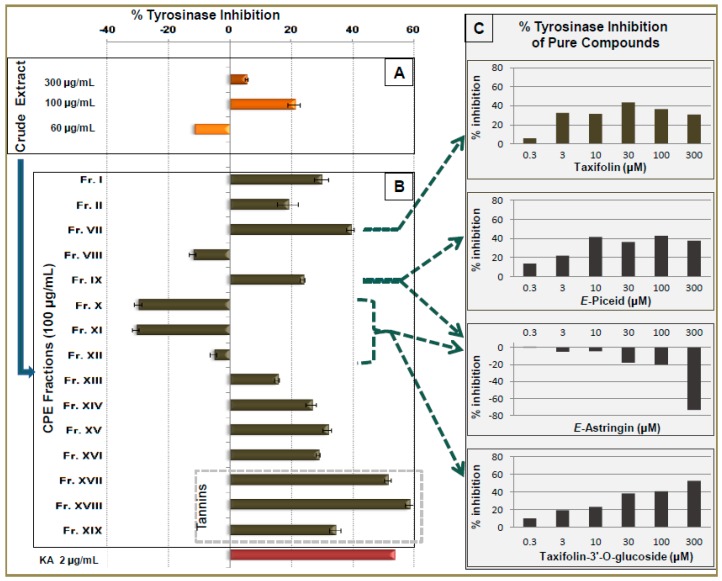
Tyrosinase inhibition activity of methanol bark extract, CPC fractions and pure compounds. (**A**) % inhibition activity of crude extract at 300, 100 and 60 μg/mL , (**B**) % inhibition activity of CPC fractions at 100 μg/mL and (**C**) % inhibition activity of pure taxifolin, *E*-piceid, *E*-astringin and taxifolin-3′-*O*-glucoside in six concentrations (0.3, 3,10, 30, 100 and 300 μM). Positive control: Kojic Acid (KA) at 2.0 μg/mL.

The tyrosinase inhibition assay showed that fractions Fr.VII, XVII and XVIII have significant inhibitory activities. The tannins enriched fractions (Fr.XVII and Fr.XVIII) were the most active with 51.7% and 58.7% inhibition at 100 μg/mL, respectively (Figure 3B).

These results are in agreement with literature data reporting tannins as potent anti-tyrosinase agents [22]. Fraction VII also presented an important anti-tyrosinase activity with 39.6% inhibition at 100 μg/mL. The main substance of this fraction, taxifolin, was further investigated regarding the anti-tyrosinase assay resulting to the significant inhibition of tyrosinase activity (43.4%) at a concentration of 30 μM (Figure 3C). These results are in accordance with the results of a recent study where taxifolin also exhibited important anti-tyrosinase activity [23]. In the same assay, Fr.X and Fr.XI presented inducing effects, on tyrosinase activity increasing the enzyme activity by 29.6% and 30.4%, respectively, at 100 μg/mL (Figure 3B). This activity is attributed to the *E*-astrigin which is the main compound in these fractions. On the other hand, Fr.IX and Fr.XII, although containing important amount of *E*-astringin, showed anti-tyrosinase effect and no effect, respectively. This may be due to the presence of other components which possess anti-tyrosinase activity and eliminate the inducing effect of *E*-astringin. In order to clarify these hypotheses, pure *E*-piceid, *E*-astringin and taxifolin-3′-*O*-glucoside were also tested. The results showed that *E*-piceid and taxifolin-3′-*O*-glucoside exhibited important anti-tyrosinase activity while *E-*astringin demonstrated significant inducing activity by increasing 73.3% the enzyme activity at a concentration of 300 μM (Figure 3C). It is important to note that although *E*-astringin and *E*-piceid have close chemical structures they exhibited opposite effect on tyrosinase assay. This indicates that a small structural difference between these two stilbene derivatives—a phenolic function on the 3′ position—is able to reverse the enzyme function. To our knowledge, this is the first report connecting *E*-astringin and taxifolin-3′-*O*-glucoside with tyrosinase activity.

### 2.5. Elastase Inhibitory Activity of CPC Fractions and Pure Compounds

**Figure 4:**
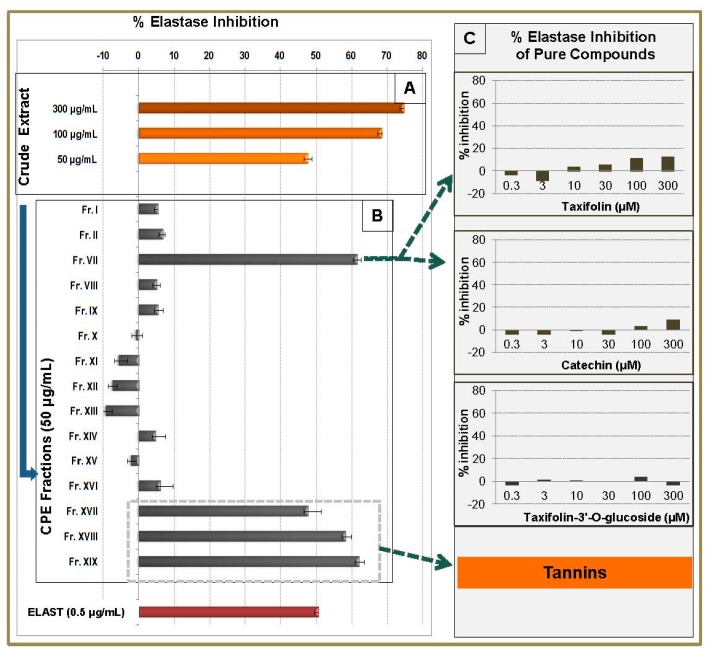
Elastase inhibitory activity of methanol bark extract, CPC fractions and pure compounds. (**A**) % inhibition activity of crude extract at 300, 100 and 50 μg/mL, (**B**) % inhibition activity of CPC fractions at 50 μg/mL and (**C**) % inhibition activity of pure taxifolin, catechin and taxifolin-3′-*O*-glucoside in six concentrations (0.3, 3,10, 30, 100 and 300 μM). Positive control: Elastatinal (ELAST) at 0.5 μg/mL.

Four of the tested CPC fractions exhibited significant inhibitory activity in our elastase assay. Fr.VII revealed an important anti-elastase activity with an inhibition value of 61.5% at 50 μg/mL (Figure 4B).

This fraction contained taxifolin as major compound, while (+)-catechin and some other minor compounds were also present (Table S1). Pure taxifolin, (+)-catechin and taxifolin 3′-*O*-gluco- pyranoside were further investigated regarding the elastase inhibitory activity. The results revealed that the three compounds have no significant effect on elastase activity (Figure 4C). This indicates that the activity of fraction VII is mainly attributed to the minor compounds contained in the mixture or to the synergistic effect of the above mentioned constituents.

Fractions XVII, XVIII and XIX (enriched in tannins) also exhibited a significant anti-elastase activity (47.6%, 58.3% and 62.1% inhibition, respectively, at 50 μg/mL), verifying the high capacity of tannins to affect the activity of numerous enzymes (Figure 4B). These results are in agreement with previous works reporting the anti-elastase activity of tannins. In a study carried out on 42 *Rosaceae* species, it was demonstrated that the extracts of high tannin content inhibited significantly the elastase activity while the less tannin-rich extracts were less active [24]. Two other studies have also shown that tannins of natural or synthetic origin inhibit the human neutrophil elastase (HNE), which plays crucial role in skin disorders such as inflamed tissue and psoriatic lesions [25,26].

### 2.6. Collagenase Inhibitory Activity of CPC Fractions and Pure Compounds

Regarding the collagenase inhibition assay, eight of the 15 CPC fractions expressed an important activity. Fr.VII was again active with an inhibition value of 52.4% at 40 μg/mL, while the activity decreased in Fr.VIII (41.3% inhibition at 40 μg/mL). These two fractions contain mainly taxifolin and (+)-catechin. More specifically, Fr.VII contains taxifolin as the main compound whereas (+)-catechin is the main constituent of Fr.VIII (Table S1). Comparing the chemical composition with the activity of the fractions, we concluded that the anti-collagenase inhibition was probably due to taxifolin while (+)-catechin was less or not active.

**Figure 5:**
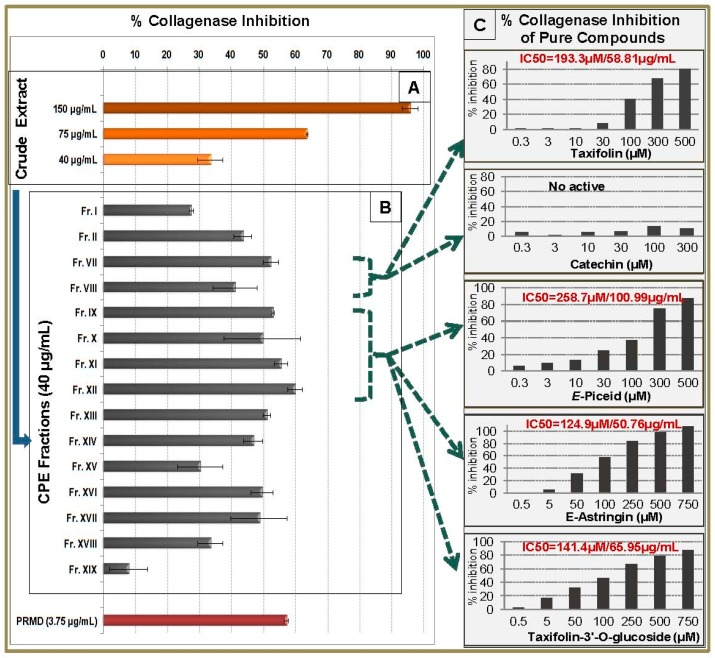
Collagenase inhibitory activity of methanol bark extract, CPC fractions and pure compounds. (**A**) % inhibition activity of crude extract at 150, 75 and 40 μg/mL, (**B**) % inhibition activity of CPC fractions at 40 μg/mL and (**C**) % inhibition activity and IC50 value of pure taxifolin, catechin, *E*-piceid, *E*-astringin and taxifolin-3′-*O*-glucoside . Positive control: Phosphoramidon (PRMD) at 3.75 μg/mL.

This was confirmed by the investigation of pure taxifolin and (+)-catechin against collagenase activity. Taxifolin exhibited significant inhibitory activity with an IC_50_ value of 193.3 μM while (+)-catechin was not active (Figure 5C). This is the first report on the anti-collagenase activity of taxifolin highlighting thus the ability of this molecule to be used as a dermo-cosmetic ingredient. Likewise, all stilbene-enriched fractions exhibited a significant anti-collagenase activity. Fraction IX, showed 53.0% inhibition while Fr.X, Fr.XI and Fr.XII (containing mainly *E*-astringin and taxifolin 3′-*O*-glucopyranoside as minor compound) exhibited 49.7%, 55.6%, and 59.9% inhibition at 40 μg/mL (Figure 5B). Due to lack of bibliographic references regarding the anti-collagenase activity of the above compounds, pure *E*-piceid, *E-*astringin and taxifolin3′-*O*-glucopyranoside were further evaluated. The result of this test showed anti-collagenase activity of the three compounds with IC50 values of 258.7 μM for *E-*piceid, 124.9 μM for *E-*astringin and 141.4 μM for taxifolin 3′-*O*-glucopyranoside. *E-*astringin, the main compound of the crude methanolic extract, was the most active of the tested compounds in the collagenase assay.

Finally, Fr.XVI and Fr.XVII, presented 49.6% and 48.7% inhibition values at 40 μg/mL, respectively. Fraction XVI contained phenolic acid derivatives, saccharose and tannins while fraction XVII contained tannins as the main compounds and saccharose. From these compounds only tannins have been reported to inhibit the collagenase activity [27].

### 2.7. In Vitro Antibacterial Activity of CPC Fractions and Pure Compounds

All CPC fractions were evaluated for their antimicrobial activity against *Staphylococcus aureus* by using a bioautography method. The results showed that fractions I, II, VII, VIII, IX, X, XI, XIII, XIV, XV, XVI and XVII have a high capacity to inhibit the microbial growth while fractions III, IV, V, VI, XII, XVIII and XIX presented low or no activity (Figure S4). Based on our chemical composition study (Table S1), dehydroabietic acid was the main compound of fractions I and II. This compound, as well as the other abietane diterpenes, are known for their antimicrobial and antifungal properties playing thus an important role in resistance of wood trees against pathogens. Fr.VII and Fr.VIII also presented significant antimicrobial activity against *Staphylococcus aureus*. The main compounds of these fractions are taxifolin (Fr.VII) and (+)-catechin (Fr.VIII). These compounds are well-known for numerous biological activities and applications in health care and cosmetic industries. Moreover the antibacterial and antifungal activities for pure compounds and plant mixtures containing taxifolin or (+)-catechin have been reported [28].

The active fractions IX–XI contain mainly stilbene derivatives. From them, fraction IX (the most active fraction as revealed in the bioautography assay) was a mixture of *E*-piceid, *E-*isorhapontin and *E*-astringin while fractions X and XI contain *E*-astringin (major compound) and taxifolin-3′-*O*-glucopyranoside (minor compound). Only a few references have reported the antimicrobial activity of these stilbene glucosides [29]. Finally, the active fractions XIII, XIV, XV, XVI and XVII contained mainly phenolic acid derivatives (fractions XIII–XV) and tannins (fractions XVI–XVII). These two categories of natural compounds are recognized for their high antibacterial and antifungal activity [30].

In order to identify the compounds responsible for the antimicrobial activity of the fractions pure taxifolin, (+)-catechin, *E*-piceid, *E-*astringin and taxifolin-3′-*O*-glucopyranose were further investigated against five pathogenic bacteria (Table 1). MIC values were determined using microdilution method and were found to range between 31.2 and 250 μg/mL. All tested compounds showed an important antibacterial activity against *Staphylococus aureus* CIP 53.154 with MIC values of 31.2 μg/mL for taxifolin and *E*-piceid, and 62.5 μg/mL for (+)-catechin, *E*-astringin, and taxifolin-3′-*O*-glucopyranoside. Moreover, *E*-astringin showed significant antimicrobial activity against *Enterococcus faecalis* ATCC 1034 (MIC 31.2 μg/mL), *Staphylococcus epidermidis* (MIC 31.2 μg/mL) and *Pseudomonas aeruginosa* ATCC 9027 (MIC 62.5 μg/mL).

## 3. Materials and Methods

### 3.1. Reagents and Materials

Methanol (MeOH), Methyl-*tert*-butyl ether (M*t*BE), ethyl acetate (EtOAc), acetonitrile (CH_3_CN) and *n-*heptane (Hept) were purchased from Carlo Erba Reactifs SDS (Val de Reuil, France). 1,1-Diphenyl-2-picrylhydrazyl (DPPH), gallic acid, ascorbic acid, taxifolin, (+)-catechin, *E*-piceid deuterated methanol (methanol-*d*_4_), and deuterated chloroform (chloroform-*d*) were purchased from Sigma-Aldrich (Saint-Quentin, France). Deionized water (H_2_O) was used to prepare all aqueous solutions. The tested microorganisms obtained from the Laboratory of microbiology (UFR Pharmacy, Reims, France) included three Gram-positive bacteria: *Enterococcus faecalis* ATCC 1034, *Staphylococcus aureus* CIP 53.154, *Staphylococcus epidermidis,* and two Gram-negative bacteria: *Escherichia coli* CIP 54.127, *Pseudomonas aeruginosa* ATCC 9027. Mueller-Hinton broth and agar medium were purchased from Biokar Diagnostics (Beauvais, France). DMSO, mushroom tyrosinase (lyophilized powder, ≥1000 units/mg solid, EC Number: 1.14.18.1), 3,4-dihydroxy-l-phenylalanine, sodium phosphate monobasic, sodium phosphate dibasic, kojic acid, elastase type IV from porcine pancreas (EC Number 254-453-6), *N*-Succinyl-Ala-Ala-Ala-*p*-nitroanilide (EC Number 257-823-5), Trizma base reagent grade, elastatinal microbial, collagenase from *Clostridium histolyticum* (released from physiologically active rat pancreatic islets Type V, ≥1 FALGPA units/mg solid, >125 CDU/mg solid, EC Number: 232-582-9), MMP 2 substrate fluorogenic, phosphoramidon disodium salt were purchased from Sigma-Aldrich.

### 3.2. Plant Material and Extraction Process

The bark of *P. abies* (2 kg) was manually collected at the trunk level (height 2 m) from trees native to Champagne-Ardenne (Champagne, France). The harvesting was held two months after felling during professional forestry activities (November 2014). After harvesting the fresh barks were placed in an oven at 30 °C for 72 h. A voucher specimen (JH-2014-9) was deposited in the Herbarium of the Botanical laboratory at the faculty of Pharmacy of Reims (University of Reims Champagne-Ardenne, Reims, France). The dry material was powdered in a mill machine and 200 g were extracted successively with *n-*heptane (E1) and methanol (E2). The extractions were performed under magnetic stirring at ambient temperature and by using 3 L of solvent each time for 24 h. After filtration, the collected solvents were evaporated to dryness and the residues were weighted, resulting in 5.0 g of *n-*heptane extract (2.5%) and 37.3 g of methanol extract (18.6%).

### 3.3. CPC Fractionation of the Methanol Extract 

CPC experiments were performed on a FCPE300^®^ apparatus (Rousselet Robatel Kromaton, Annonay, France) equipped with a rotor made of seven circular partition disks containing 231 partition twin cells (~1 mL per cell, 303.5 mL total column capacity) and connected to a Preparative 1800 V7115 pump (Knauer, Berlin, Germany). The system was coupled to a UVD 170S detector set at 210, 254, 280, and 366 nm (Dionex, Sunnyvale, CA, USA). Fractions of 20 mL were collected by a Superfrac collector (Pharmacia, Uppsala, Sweden).

The crude extract was fractionated by using the triphasic solvent system Hept/M*t*BE/CH_3_CN/H_2_O 1/1/1/1 (*v*/*v*/*v*/*v*) in a step-gradient elution mode. The solvent system was prepared as described previously [13]. The column was filled with the lower phase (stationary phase) in the ascending mode at a flow rate of 50 mL/min and 200 rpm in order to replace the stock solution (MeOH/H_2_O, 1/1, *v*/*v*). Then the sample (3 g) diluted in a solution of 15 mL lower phase/10 mL middle phase/5 mL upper phase was injected into the column via a 35 mL sample loop. The rotation speed was set at 1000 rpm. The mobile phase I (MPI) was pumped starting with the flow rate of 1 mL/min and increasing gradually to 20 mL/min during the first 5 min. After that the flow rate remained stable at 20 mL/min during the whole experiment. The MPI was pumped for the first 40 min and then was changed to MPII for the next 58 min (elution mode). The experiment was completed by pumping the stationary phase in ascending mode at 20 mL/min and 1000 rpm (extrusion step). After equilibration of the column the final stationary phase retention was calculated at 62%.

The separation process was monitored by UV at 254 nm and the total process duration was 125 min. All collected fractions were spotted on Merck TLC plates (Darmstadt, Germany) coated with silica gel 60 F254 and developed with toluene/ethyl acetate/acetic acid/formic acid (30:70:11:11, *v*/*v*/*v*). After detection at UV254 and UV366, the plates were sprayed with vanillin–sulfuric acid and heated to 100 °C for 5 min. Fractions of similar composition were combined resulting in 19 final fractions (Fr.I–Fr.XIX, Figure S1, TLC chromatogram of combined CPC fractions). The resulting fractions were dried under vacuum and weighted (Table S1).

### 3.4. NMR Chemical Profiling of the CPE-Generated Fractions

An aliquot of each fraction from Fr.I to Fr.XIX (20 mg) was dissolved in 600 µL methanol-*d_4_*. NMR analyses were performed at 298 K on an Avance AVIII-600 spectrometer (Bruker, Karlsruhe, Germany) equipped with a cryoprobe optimized for ^1^H detection and with cooled ^1^H, ^13^C and 2D coils and preamplifiers. ^13^C-NMR spectra were acquired at 150.91 MHz. A standard zgpg pulse sequence was used with an acquisition time of 0.909 s and a relaxation delay of 3 s. For each sample, 1024 scans were co-added to obtain a satisfactory signal-to-noise ratio. The spectral width was 238.9070 ppm and the receiver gain was set to the highest possible value. A 1 Hz line broadening filter was applied to each FID prior to Fourier transformation. The spectra were manually phased and baseline corrected using the TOPSPIN 3.2 software (Bruker) and calibrated on the central resonance (δ 49.1 ppm) of methanol-*d*_4_. The ^13^C-NMR spectra are provided as supplementary data (Figure S2). A minimum intensity threshold of 0.3% (relative to the most intense signal of each spectrum) was then used to automatically collect all positive ^13^C-NMR signals while avoiding potential noise artifacts. Each peak list was then converted into a text file. Absolute intensities of the collected peaks in the fraction series were aligned by using an in-house algorithm written in the python language. The principle was to divide the ^13^C spectral width (from 0 to 200 ppm) into regular bins, i.e. chemical shift intervals (Δδ = 0.2 ppm), and to associate the absolute intensity of each ^13^C peak to the corresponding bin. The bins for which no signal was detected in any fraction were removed from the bin list. The resulting table was imported into the PermutMatrix version 1.9.3 software (LIRMM, Montpellier, France) for clustering analysis on raw peak intensity values. The classification was performed on the rows only, i.e. on the chemical shift bins. The Euclidian distance was used to measure the proximity between samples and the Ward′s method was performed to agglomerate the data. The resulting ^13^C chemical shift clusters were visualized as dendrograms on a two-dimensional map. The higher the intensity of ^13^C-NMR peaks, the brighter the color on the map.

In parallel, a literature survey was performed to obtain names and structures for a maximum of *P. abies* metabolites already described in the literature. In total, 42 metabolites were added to a locally built ^13^C-NMR chemical shift database (ACD/NMR Workbook Suite 2012 software, ACD/Labs, Toronto, ON, Canada) comprising the chemical shifts and structures of ≈ 1900 structures of natural products. Structures were drawn with ACD/Labs ChemSketch and their predicted chemical shifts were assigned to the corresponding carbon positions. For metabolite identification, each ^13^C chemical shift cluster obtained from HCA was submitted to the structure search engine of the database management software. A ^13^C-NMR chemical shift tolerance of ±2 ppm was used.

Additional 2D-NMR experiments (HSQC, HMBC, and COSY) of fractions Fr.IV, Fr.IX, Fr.XV, and Fr.XVI, were performed on the same Bruker Avance AVIII-600 spectrometer, by using standard Bruker pulse programs in order to elucidate the structures of some minor compounds.

The structures of the identified compounds were verified by ESI(−)-HRMS analysis while for purity purposes LC-ESI(−)-HRMS data were used. The analysis was performed on a hybrid LTQ-Orbitrap Discovery Mass Spectrometer (Thermo Scientific, Bremen, Germany). The mass spectrometer was equipped with electrospray ionization (ESI) source, operated in negative mode (LC-HRMS data and HRMS spectra in Supplementary Materials).

### 3.5. Recovery of Pure E-Astringin from the CPE-Generated Fractions

The purification of *E*-astringin was achieved by using Sephadex LH-20 column chromatography. 150 mg of Fr.XI (diluted in 0.5 mL MeOH) were subjected to CC (Sephadex^®^ LH-20, Sigma-Aldrich Chemie GmbH, Taufkirchen, 10 mm × 285 mm,) and eluted with MeOH. All obtained fractions were initially analyzed by TLC and those of similar chemical content were combined giving finally four main fractions (Fr.A–D). Fractions B (24.2 mg) and D (100.5 mg) contained taxifolin-3′-*O*-glucoside and *E*-astringin respectively, both in high purity (>95%). (Figure S2). The purity of isolated compounds was determined by LC-HRMS and ^1^H-NMR analysis.

### 3.6. Antioxidant Activity-DPPH Radical Scavenging Assay

The DPPH radical scavenging assay was performed according to a previously described method [31] with slight modifications. The stock DPPH solution (314 μΜ) was prepared by diluting 12.4 mg in 100 mL absolute ethanol, vortexed and kept in dark at room temperature until its use. Gallic acid was used as a positive control at a concentration of 29.4 µM. The crude bark extract was diluted in DMSO at final concentrations of 200, 50 and 25 μg/mL while CPC fractions were diluted in DMSO at final concentrations of 25 μg/mL. In a microwell plate, 190 μL of the DPPH solution and 10 μL of gallic acid or samples were added. When DPPH reacts with an antioxidant compound, which can donate hydrogen, it is reduced. The changes in color (from deep violet to light yellow) were read (Absorbance (Abs)) at 517 nm after 30 min of incubation in the dark at room temperature using the reader Infinite 200 PRO series (Tecan Group Ltd., Männedorf, Switzerland). Experiments were performed in triplicate for each sample and twice in total A negative control containing 10 μL DMSO and 190 μL DPPH was performed each time. Blanks contained 190 μL EtOH and 10 μL sample. The radical scavenging activity percentage (AA%) was determined as follow: AA% = [1 − ((A_sample_ − A_blank_)/A_control_)] × 100, where A_control_ is the absorbance of the negative control, A_sample_ is the absorbance after the reaction of samples with DPPH, and A_blank_ is the absorbance of samples with EtOH instead of DPPH.

### 3.7. Whitening Activity-Tyrosinase Inhibition Assay

Crude extract was evaluated at concentrations of 300, 100 and 60 μg/mL, CPC fractions at 100 μg/mL (final concentration in the well), while pure compounds were evaluated at six concentrations; 0.3, 3, 10, 30, 100 and 300 μM. The capacity of the samples to inhibit the catalytic action of tyrosinase in the oxidation of l-DOPA to dopachrome was determined by an enzymatic method described by Masuda et al. [32] with some modifications. Tyrosinase activity was measured at 475 nm using the reader Infinite 200 PRO series (Tecan). The inhibitory potency of the samples against this enzyme was compared with this of positive control, kojic acid (IC_50_ = 14 μM) known as strong tyrosinase inhibitor. In a 96-well microplate, 80 μL of sodium phosphate buffer (PBS) (1/15 M, pH = 6.8), 40 μL of the tested sample (dissolved in the PBS buffer from 1.5 to 0.3 mg/mL) and 40 μL of mushroom tyrosinase 92 U/mL were mixed and incubated for 10 min at room temperature avoiding light exposure. Afterwards, 40 μL of 2.5 mM L-DOPA dissolved in buffer were added and the mixture was incubated for 5 min before measurement of dopachrome formation at 475 nm. Experiments were performed in triplicates and twice in total. The final DMSO concentrations did not exceed 3% of total volume. The inhibition percentage was calculated as followed: Inhibition (%) = [((A_control_ − A_control′s blank_) – (A_sample_ − A_sample′s blank_))/(A_control_ − A_control′s blank_)] × 100, where A_control_ is the absorbance of the mixture consisting of buffer, tyrosinase, sample solvent and substrate and A_sample_ is the absorbance of the mixture of buffer, tyrosinase, samples or kojic acid solution and substrate. Blanks contained all the above mentioned components except the enzyme.

### 3.8. Elastase Inhibition Assay

The porcine pancreatic elastase type IV (PPE), a lyophilized powder at ≥4 units/mg protein, was used for this bioassay. Crude extracts were evaluated at concentrations of 300, 100 and 50 μg/mL, CPC fractions at 50 μg/mL (final concentration in the well) while pure compounds were evaluated at six concentrations; 0.3, 3, 10, 30, 100 and 300 μM. PPE inhibition was tested spectrophotometrically according to a previously described method with slight modifications [33], using *N*-succinyl-Ala-Ala-Ala-*p*-nitroanilide as substrate, and monitoring the release of *p*-nitroaniline. The amount of *p*-nitroaniline was determined by measuring the absorbance at 405 nm. The reaction mixture initially contained 70 μL Trizma-base buffer (50 mM, pH = 7.5), 10 μL of sample (500 μg/mL in buffer) and 5 μL of elastase (0.4725 U/mL). The resulting solutions were incubated for 15 min at room temperature avoiding light exposure. Afterwards, 15 μL of 2 mM *N*-succinyl-Ala-Ala-Ala-*p*-nitroanilide dissolved in Trizma buffer were added and the mixtures were incubated for 30 min at 37 °C. Elastatinal which is a strong irreversible competitive inhibitor of PPE was used as a positive control (IC_50_ = 0.5 μg/mL). Experiments were performed in triplicate for each sample and twice in total. Absorbances were measured using the reader Infinite 200 PRO series (Tecan). The inhibition percentage of elastase was calculated by the formula in Section 3.7, where A_control_ is the absorbance of the mixture containing the buffer, elastase, the sample solvent and the substrate, and A_sample_ is the absorbance of the buffer, elastase, sample or elastatinal and substrate mixture. Blank experiments were performed for each sample with all the reagents except the enzyme.

### 3.9. Collagenase Inhibition Assay

The anti-collagenase activity of crude extracts, CPC fractions and pure compounds was determined based on a previously described spectrofluorimetric method with slight modifications [34]. In a 96-well microtiter plate, 25 μL of Tris-HCl buffer (10 mM, pH = 7.3), 25 μL of sample (dissolved in Tris-HCl buffer (<2% DMSO)) and 25 μL of collagenase from *Clostridium histolyticum* (100 μg/mL in Tris-HCl buffer) were preincubated for 10 min at 37 °C. Afterwards, 25 μL of MMP2 substrate (MCA-Pro-Leu-Ala-Nva-DNP-Dap-Ala-Arg-NH_2_) solution in buffer at an initial concentration of 50.8 μM were added. The fluorescence values (fluorescent intensity) were measured at an excitation maximum of 320 nm and an emission maximum of 405 nm after 30 min incubation at 37 °C using a fluorescence plate reader (Galaxy fluo star, MTX Lab Systems, Bradenton, FL, USA Light exposure was avoided during the incubation periods. Phosphoramidon, a metallo-endopeptidase inhibitor, was used as a positive control (IC_50_ = 6.9 μΜ). Crude extract was evaluated at concentrations of 150, 75 and 40 μg/mL, CPC fractions at 40 μg/mL while pure compounds were evaluated at seven concentrations; 0.3, 3, 10, 30, 100, 300 and 500 μM. All assays were performed in triplicate and twice in total. The inhibition percentage was calculated as follows: Inhibition (%) = [((F_control_ − F_control′s blank_) – (F_sample_ − F_sample′s blank_))/(F_control_ − F_control′s blank_)] × 100, where F_control_ is the fluorescence of buffer, collagenase, sample solvent and substrate and F_sample_ is the absorbance of buffer, collagenase, sample or phosphoramidon and substrate. Blanks contained all the components except the enzyme. For the active pure compounds the IC50 values were calculated.

### 3.10. Antimicrobial Assay

#### 3.10.1. Bioautography

The antibacterial activity of the crude methanol extract and CPC fractions (Fr.I toFr.XIX) was determined by an immersion bioautography method [35]. An aliquot of each sample (20 mg) was solubilized in 1 mL of methanol and the resulting solutions were spotted onto Merck 60 F254 pre-coated silica gel plates (10 cm × 10 cm). Gentamicin (50 µg) was also spotted on the plates as a positive control. The TLC plates were directly dried without migration and sterilized. The plates were then covered by Mueller-Hinton (MH) agar medium containing a *Staphylococcus aureus* CIP 53.154 suspension (10^5^ bacteria/mL) in square Petri dishes. After incubation at 37 °C for 24 h, bacterial growth was revealed by a 2 mg/mL solution of thiazolyl blue tetrazolium bromide (MTT) and growth inhibition zones were measured manually. White stains indicated where reduction of MTT to the colored formazan did not take place due to the presence of extracts that inhibited bacterial growth. Solvents were also checked for absence of antibacterial activity.

#### 3.10.2. MIC Determination

The pure compounds were tested against five pathogenic bacteria while the MIC values were determined by broth microdilution method (liquid medium). The bacteria were incubated overnight at 37 °C in tubes containing Mueller-Hinton (MH) broth medium. Then, the bacteria were diluted with MH-broth to 10^5^ bacteria/mL by means of serial dilution just before the antimicrobial assays. A serial dilution technique using 96-well microliter plates was used to determine the MIC values. Nine concentrations of plant extract, from 500 µg/mL to 1.9 µg/mL, were used. Two wells were represented as bacteria culture control (positive control) and medium sterility control (negative control). Then the wells were loaded with MH liquid medium and bacterial suspension (10^5^ bacteria/mL) giving a final volume of 200 μL. The plates were incubated overnight at 37 °C. Bacterial growth was observed visually and then after direct spray of 0.2 mg/mL MTT to each well and incubation at 37 °C for at least 30 min. Bacterial growth was indicated by a violet color. MIC values were determined as the lowest concentrations of compounds showing an inhibition on bacteria growth (clear wells). This test was performed in duplicate.

## 4. Conclusions

In the present work the methanol extract of common spruce bark was investigated regarding the chemical composition, separation and biological evaluation of the generated fractions and pure compounds. The bio-guided fractionation of this extract led to the recovery of high amount of *E-*astringin, to be precise 0.93 g in a single run of 125 min (31% yield from the crude extract). This compound was found to induce significantly the tyrosinase activity (increasing 73.3% the enzyme activity at 300 μM) and showed important anti-collagenase activity (IC50 value of 258.7 μM). Moreover, *E-*astringin demonstrated significant antimicrobial activity against *Enterococcus faecalis* ATCC 1034 (MIC 31.2 μg/mL), *Staphylococcus epidermidis* (MIC 31.2 μg/mL), *Staphylococcus aureus* CIP 53.154 (MIC 62.5 μg mL^−1^) and *Pseudomonas aeruginosa* ATCC 9027 (MIC 62.5 μg/mL). In parallel, taxifolin and taxifolin-3′-*O*-glucoside were found to be active components of the extract with significant anti-tyrosinase activity, anti-collagenase activity (IC50 at 193.3 μM and 141.4 μM respectively) and important antibacterial activity against *Staphylococcus aureus* CIP 53.154 (MIC values of 31.2 μg/mL and 62.5 μg/mL respectively). These results offer new perspectives for dermo-cosmetic applications of these bioactive molecules expanding thus the industrial exploitation of common spruce bark.

## Figures and Tables

**Table 1:** Antimicrobial activity of pure taxifolin, (+)-catechin, *E*-piceid, *E*-astringin and taxifolin-3′-*O*-glucopyranose against five pathogenic bacteria (liquid medium). MIC values of 31.2 μg/mL and 62.5 μg/mL correspond to important antibacterial activity (red values in the table).

Microorganisms	MIC (μg/mL)				
	Taxifolin	Catechin	*E*-Piceid	*E*-Astringin	Tax-3’-*O*-gluc
Gram positive bacteria					
*Enterococcus faecalis ATCC 1034*	125	125	125	31.2	125
*Staphylococcus aureus CIP 53.154*	31.2	62.5	31.2	62.5	62.5
*Staphylococcus epidermidis*	250	125	125	31.2	125
Gram negative bacteria					
*Escherichia coli CIP 54.127*	250	250	250	125	250
*Pseudomonas aeruginosa ATCC 9027*	125	125	125	62.5	125

## Supplementary Materials

The following are available online at www.mdpi.com/1420-3049/21/11/1586/s1.
