# Supplementary material for: Bio-Guided Isolation of Methanol-Soluble Metabolites of Common Spruce (Picea abies) Bark by-Products and Investigation of Their Dermo-Cosmetic Properties
Source: Molecules. 2016 Nov 21;21(11):1586. doi: 10.3390/molecules21111586 (PMC6272914; doi:10.3390/molecules21111586)
Supplement: Supplementary file 1 [file molecules-21-01586-s001.pdf]

Supplementary Materials: Bio-Guided Isolation of Methanol Soluble Metabolites of Common Spruce (*Picea abies*) Bark by-Products and Investigation of Their Dermo-Cosmetic Properties

Apostolis Angelis, Jane Hubert, Nektarios Aligiannis, Rozalia Michalea, Amin Abedini, Jean-Marc Nuzillard, Sophie C. Gangloff, Alexios-Leandros Skaltsounis and Jean-Hugues Renault

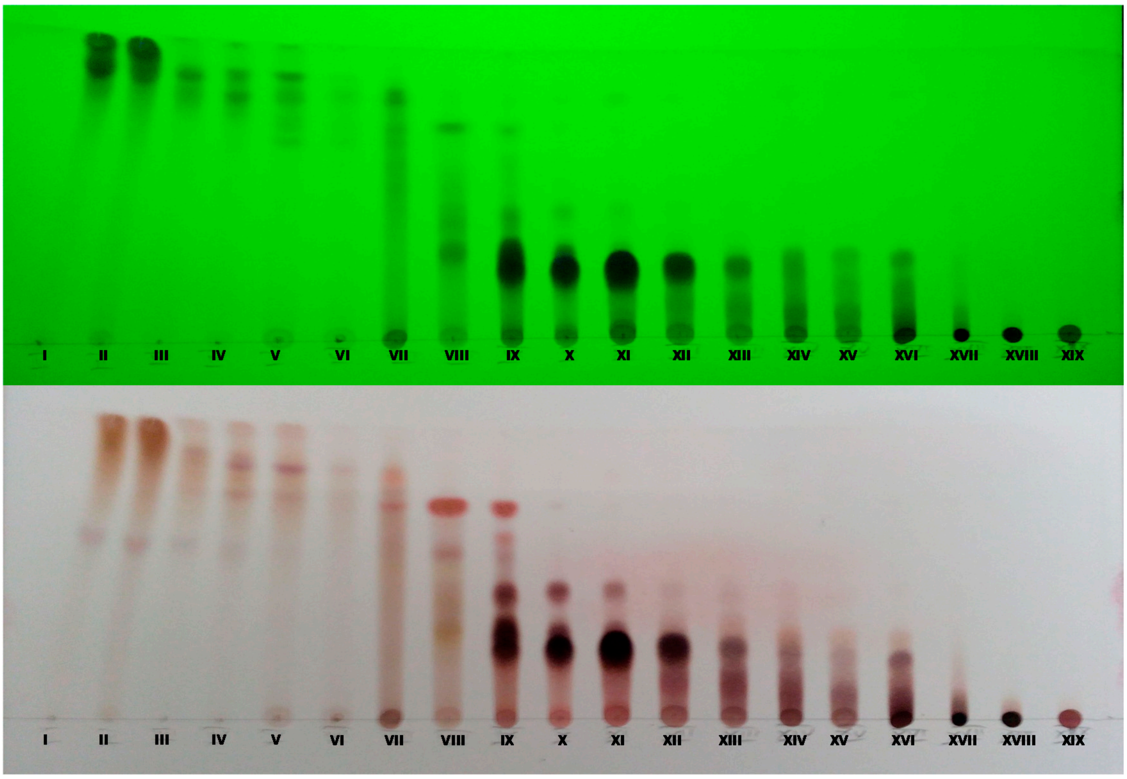

Figure S1. TLC chromatogram of the compared CPC fractions under UV lamb (254 nm **top**) and after spray with vanillin/H_2SO_4/MeOH solution and heating (**below**).

Table S1. The weights and chemical composition of CPC fractions of *P. abies* bark extract.

Fractions	Weight (mg)	Chemical Composition	Fractions	Weight (mg)	Chemical Composition
I	65	Comp1/FC	XI	420	Comp8(M)/Comp9
II	210	Comp1/FC	XII	90	Comp8(M)/Comp9
III	10	Comp2/AD/FC	XIII	75	Comp8/Comp 9/Comp 10
IV	10	Comp2/Comp3/UFA	XIV	45	Comp10/Comp 11/Comp 12
V	13	Comp3/ UFA	XV	40	Comp10/Comp 11/Comp 12/Comp 13
VI	18	UFA	XVI	120	Comp 12/Comp13/Comp14/Tannins
VII	80	Comp4 (M)/ Comp5/MC	XVII	900	Comp14/Tannins(M)
VIII	90	Comp4/Comp5 (M)/MC	XVIII	300	Comp14 + Tannins(M)
IX	225	Comp6/ Comp7/ Comp8	XIX	30	Tannins
X	425	Comp7/Comp8(M) /Comp9			

FC = Fatty compounds, AD = Abietane diterpenes, UFA = Unsaturated Fatty Acid, MC = Minor Compounds, (M) = major compound.

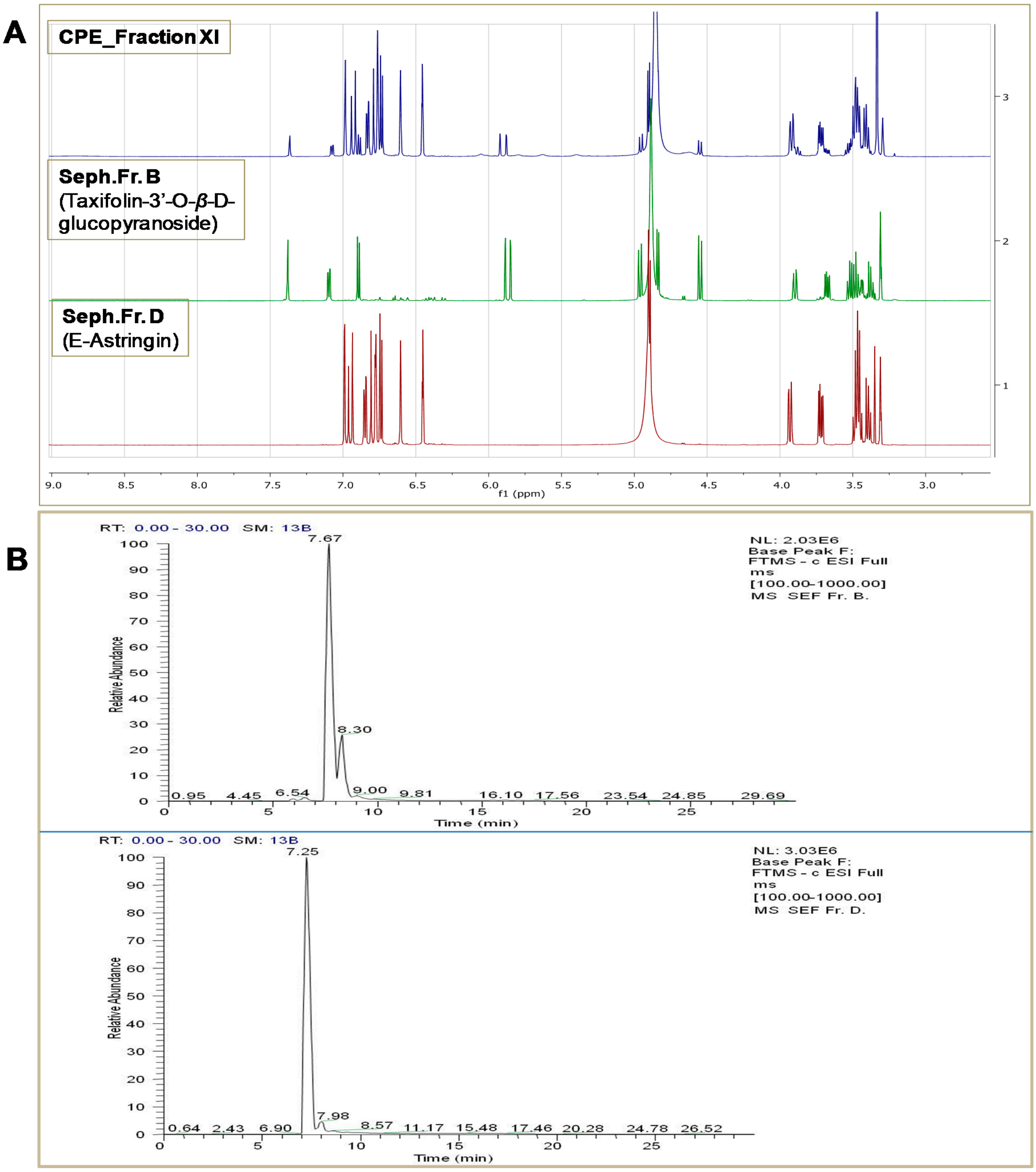

Figure S2. ^{1}H-NMR spectra (**A**) and LC-ESI(-)-HRMS spectra (**B**) of *E*-astringin and taxifolin-3′-O-*β*-D-glucopyranoside obtained from the analysis of CPC_Fr. XI by Sephadex LH-20 Column Chromatography

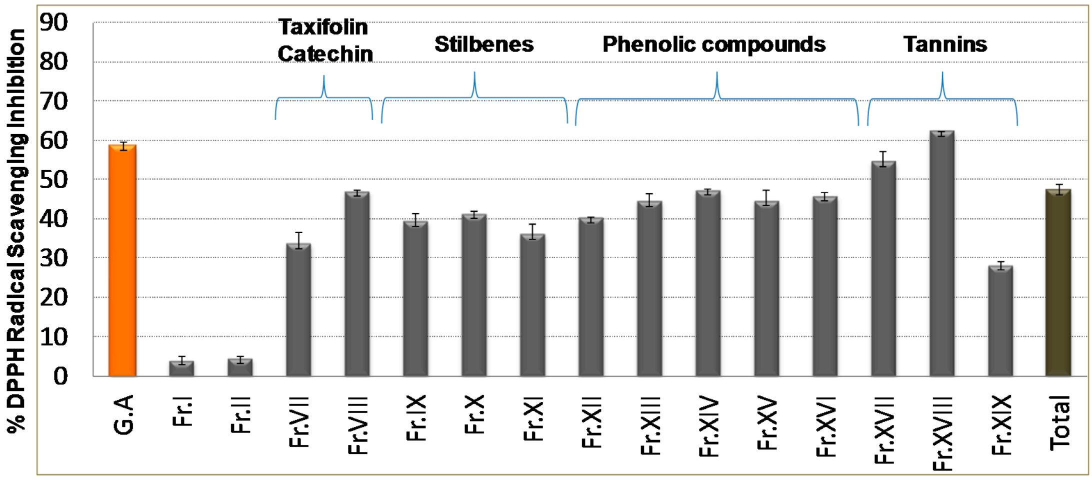

Figure S3. % DPPH Inhibition of CPC fractions at 25 μg/mL. GA: Gallic Acid (at 5.0 μg/mL), Total: Crude Methanolic Extract from bark of *P. abies* (at 25 μg/mL).

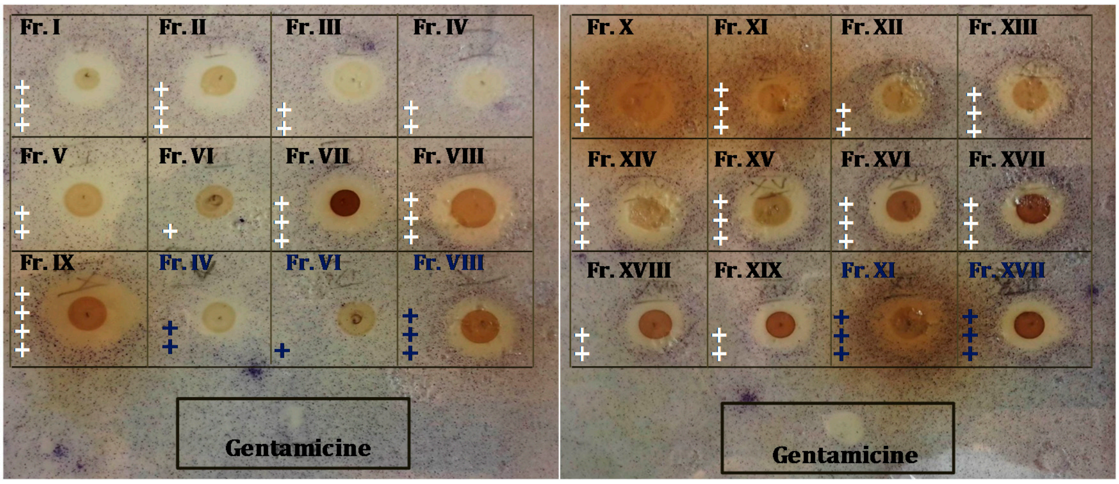

Figure S4. Antimicrobial activity of CPC fractions against *Staphylococcus aureus* by using bioautographic method. (+): low activity, (++): middle activity, (+++): high activity, (++++): very high activity). Fractions IV, VI, VIII, XI and XVII were spotted twice in the TLC plate (blue color) in order to verify the accuracy of the method

ESI(-)-HRMS Spectra of Identified Compounds

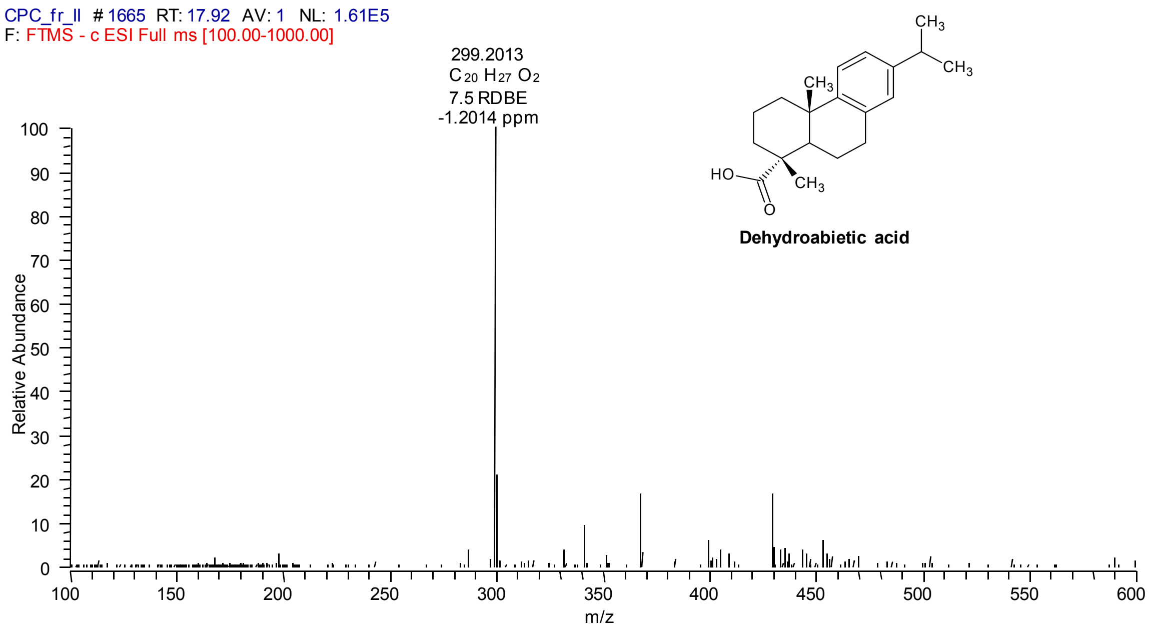

Figure S5. ESI(-)-HRMS spectrum of dehydroabietic acid.

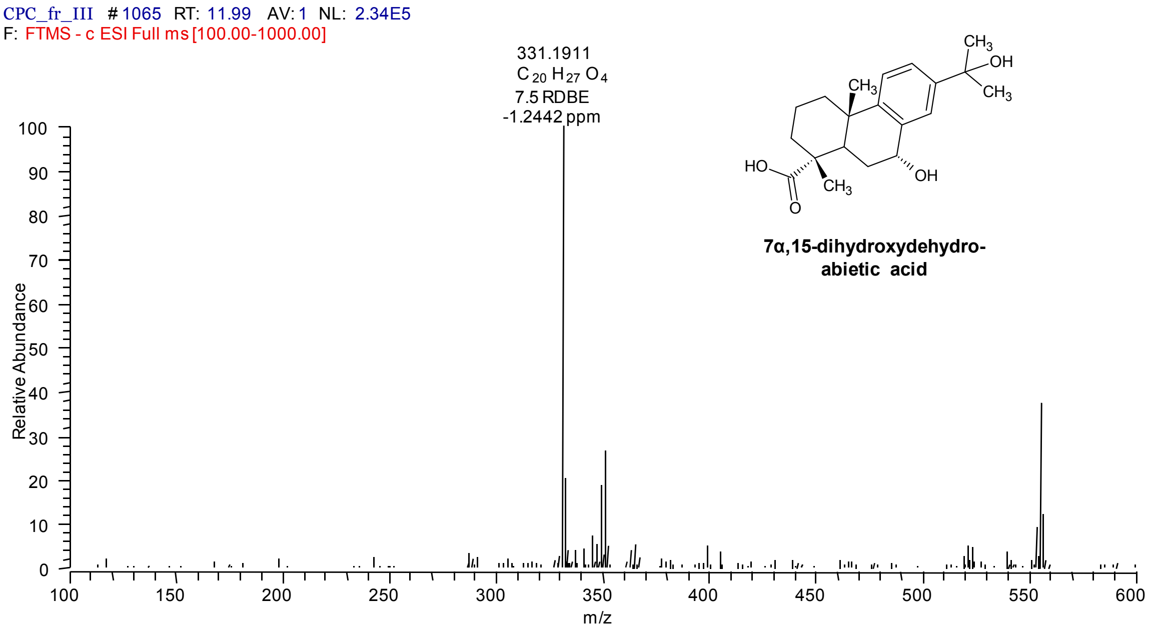

Figure S6. ESI(-)-HRMS spectrum of 7a,15-dihydroxydehydroabietic acid.

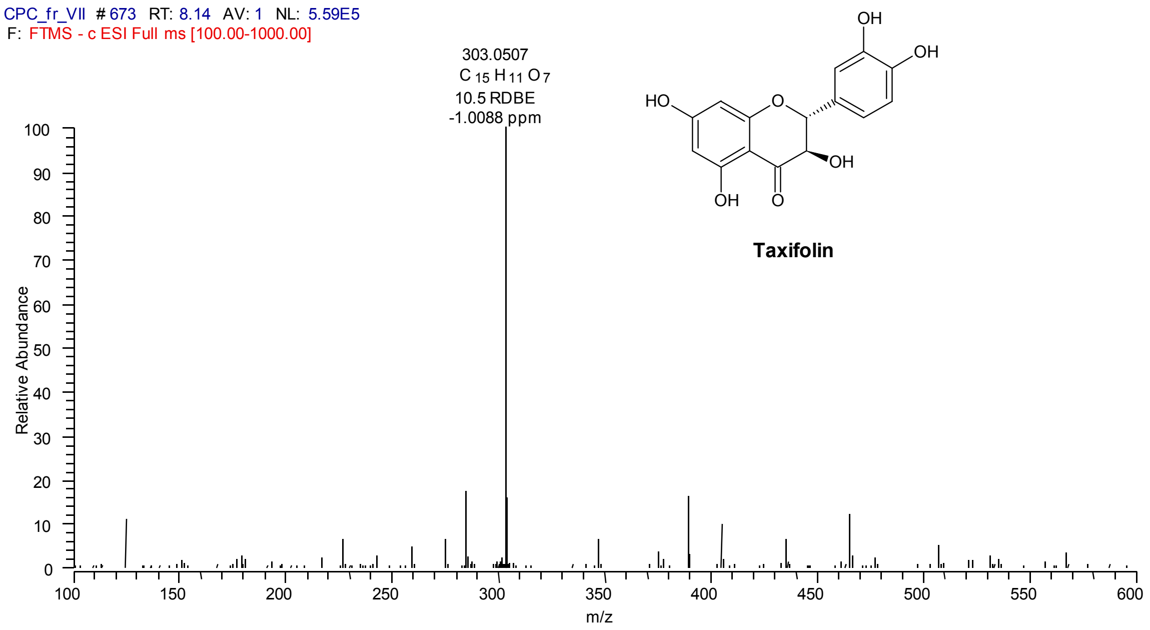

Figure S7. ESI(-)-HRMS spectrum of taxifoline.

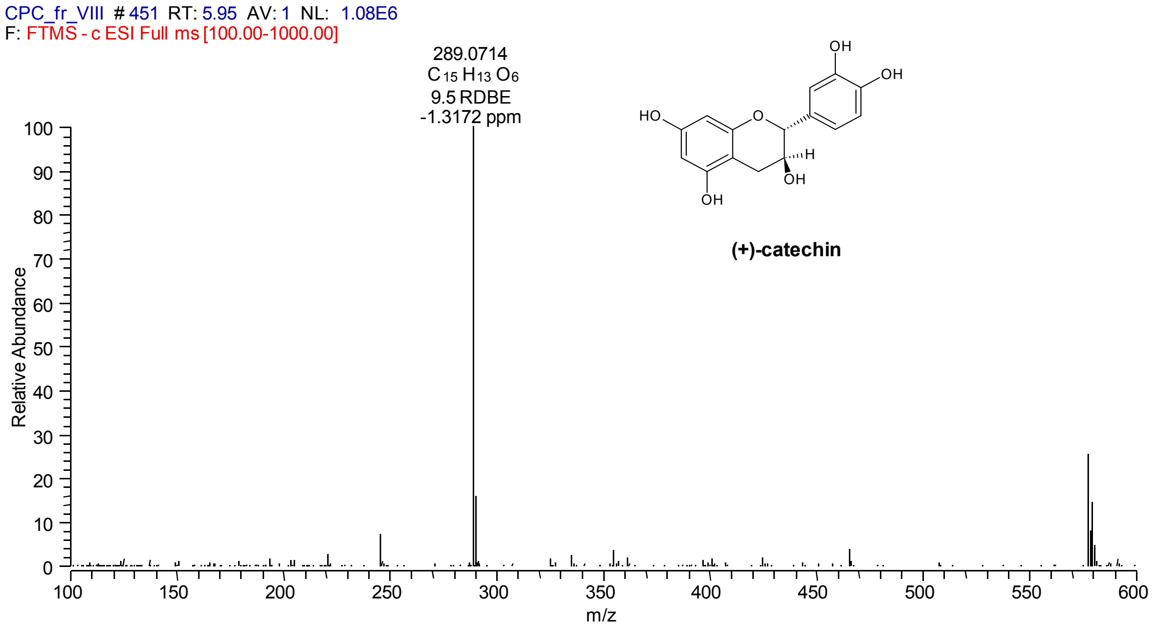

Figure S8. ESI(-)-HRMS spectrum of (+)-catechin.

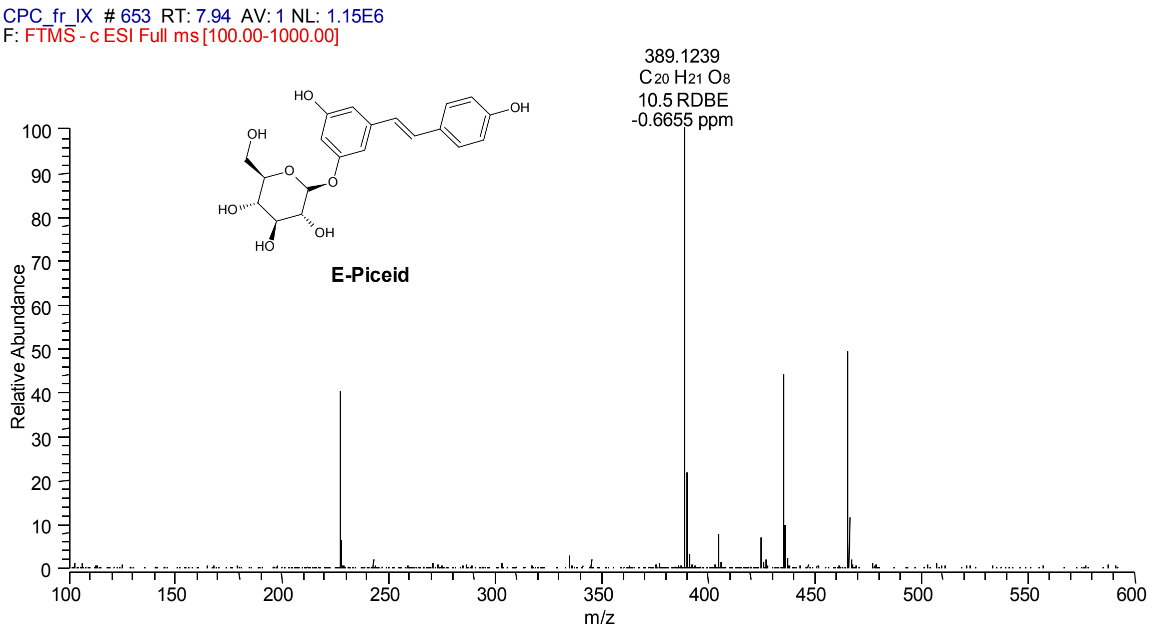

Figure S9. ESI(-)-HRMS spectrum of *E*-piceid.

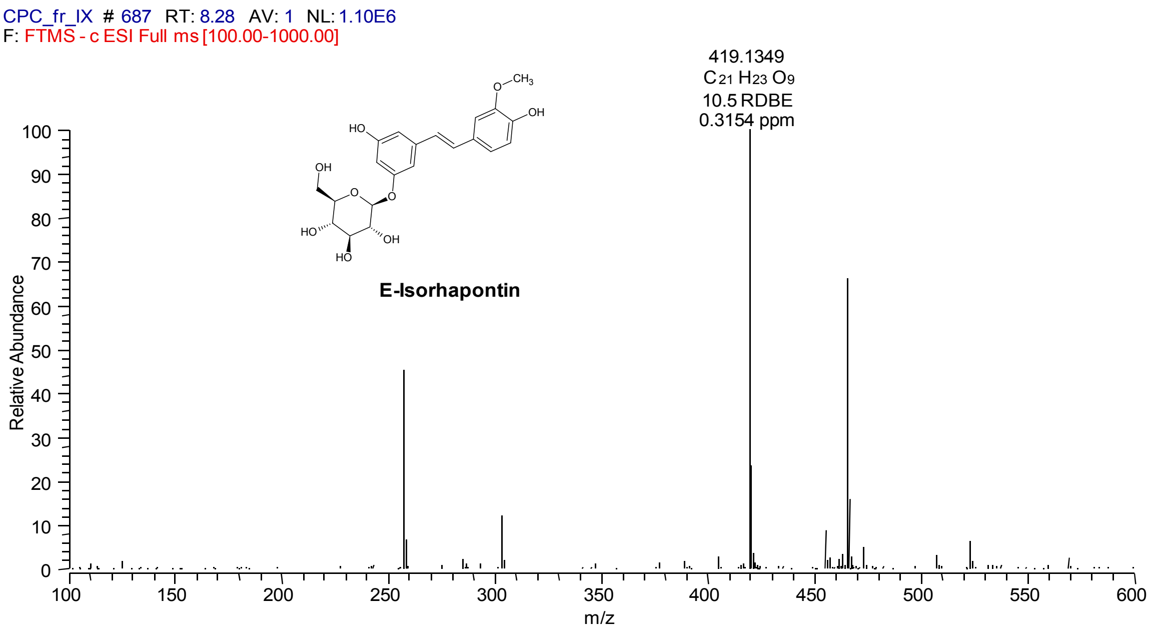

Figure S10. ESI(-)-HRMS spectrum of *E*-isorhapontin.

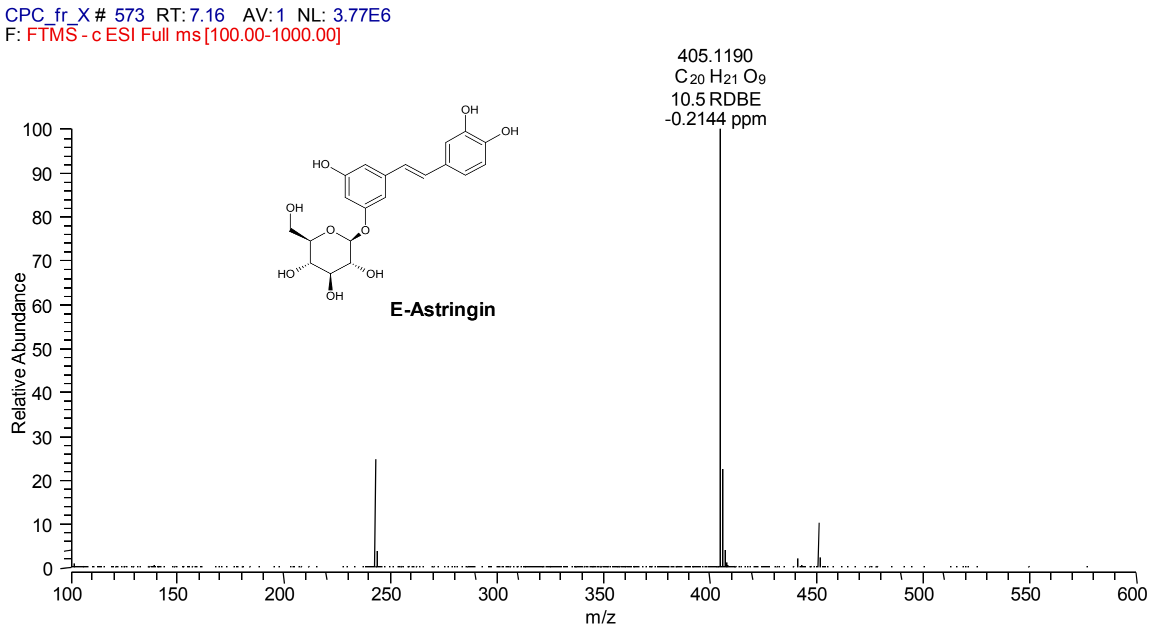

Figure S11. ESI(-)-HRMS spectrum of E-astringin.

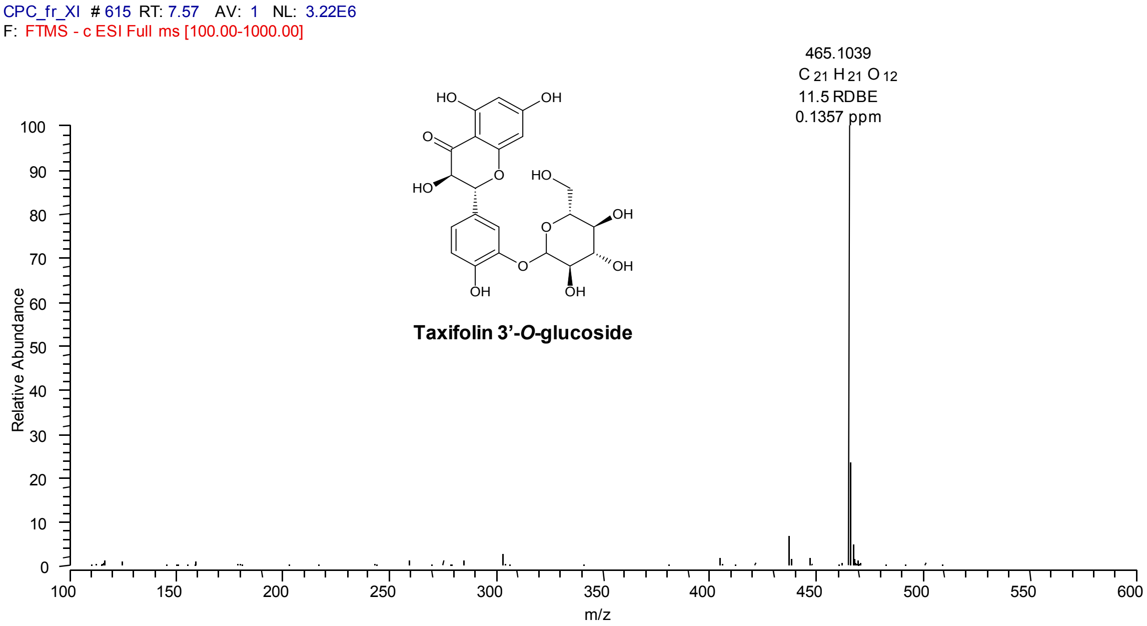

Figure S12. ESI(-)-HRMS spectrum of taxifoline-3′-*O*-*β*-D-glucopyranoside.

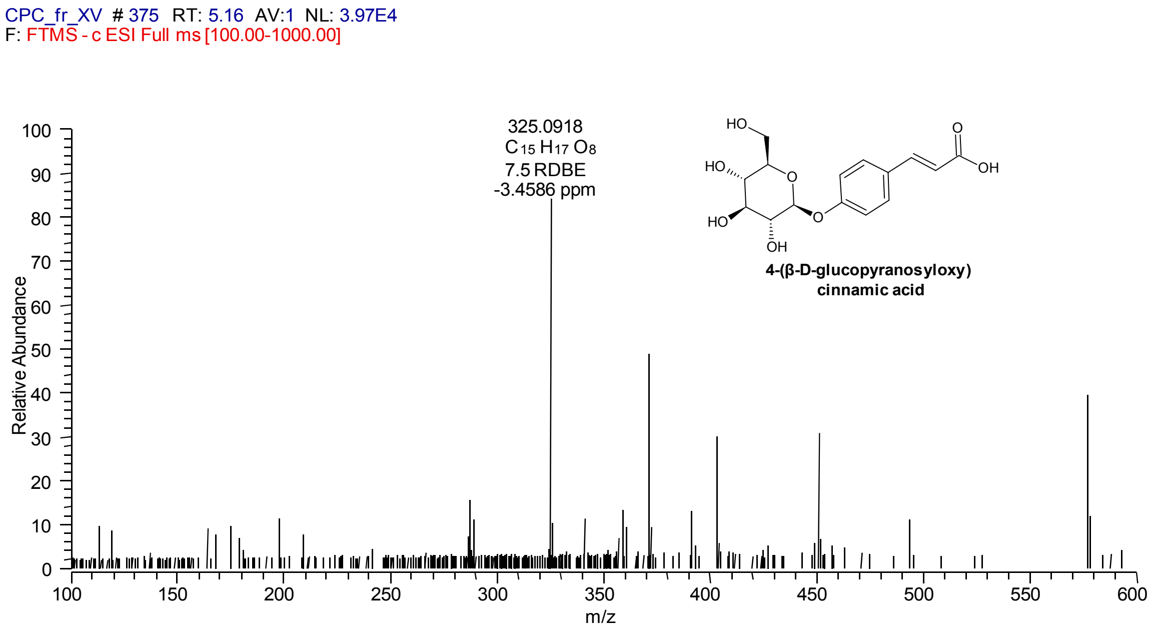

Figure S13. ESI(-)-HRMS spectrum of 4-(*β*-D-glucopyranosyloxy)cinnamic acid.

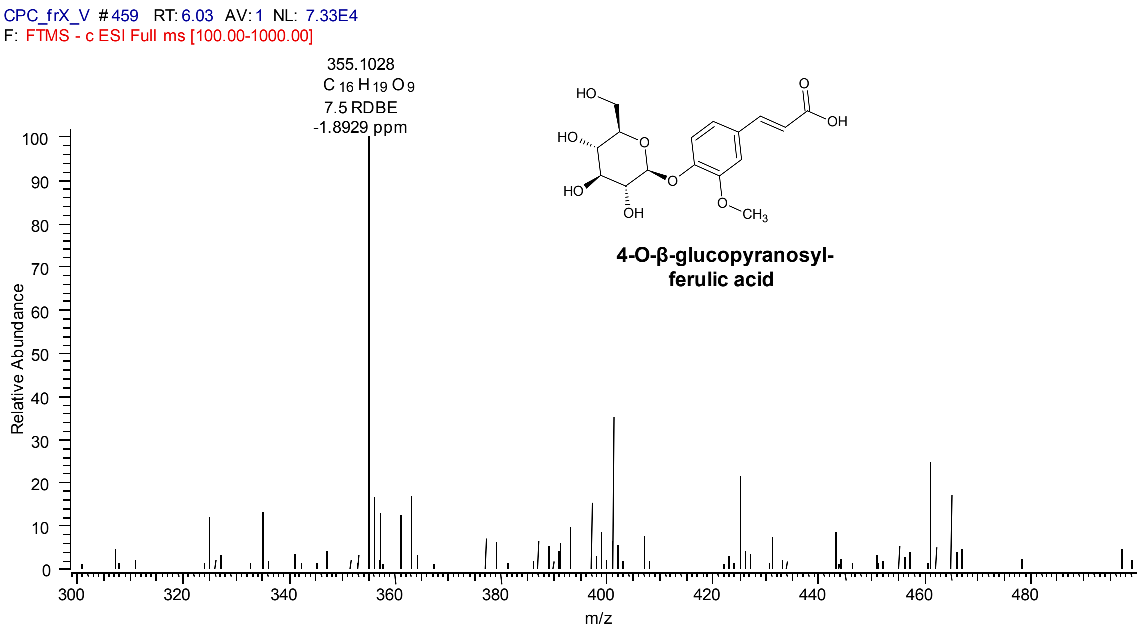

Figure S14. ESI(-)-HRMS spectrum of 4-*O*-β-glucopyranosyl- ferulic acid.

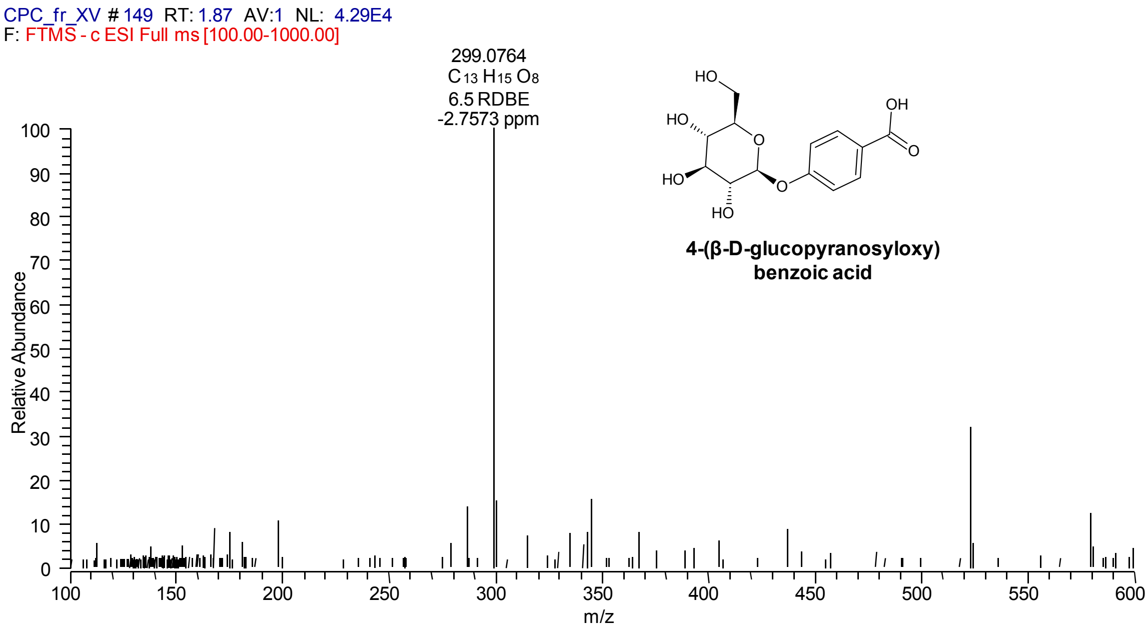

Figure S15. ESI(-)-HRMS spectrum of 4-(β-D-glucopyranosyloxy)benzoic acid.

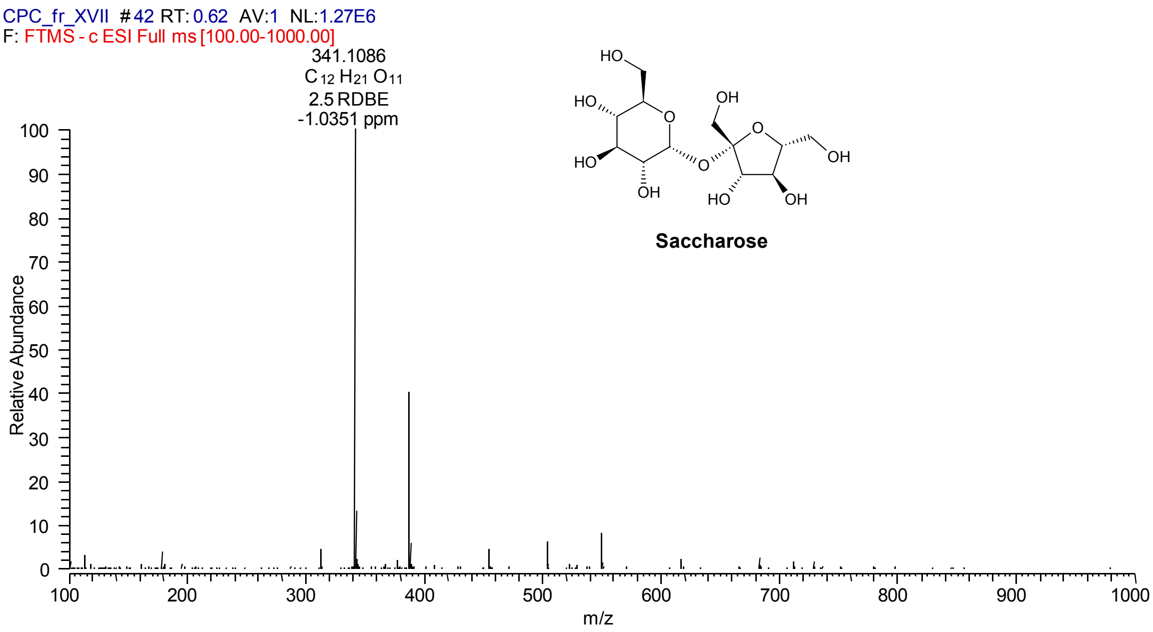

Figure S16. ESI(-)-HRMS spectrum of saccharose.
